# Safe Coated Microneedles with Reduced Puncture Occurrence after Administration

**DOI:** 10.3390/mi11080710

**Published:** 2020-07-22

**Authors:** Hye-Rin Jeong, Hyesun Jun, Hye-Ran Cha, Jae Myun Lee, Jung-Hwan Park

**Affiliations:** 1 Department of Bionano Technology, Gachon University, Gyeonggi-do 13120, Korea; lyn0540@gmail.com; 2 QuadMedicine R&D Centre, QuadMedicine, Inc., Seongnam 13209, Korea; stella@quadmedicine.com; 3 Department of Microbiology and Immunology, Institute for Immunology and Immunological Diseases, Brain Korea 21 PLUS Project for Medical Science, Yonsei University College of Medicine, Seoul 03722, Korea; HRCHA@yuhs.ac (H.-R.C.); JAEMYUN@yuhs.ac (J.M.L.)

**Keywords:** puncture performance, coated microneedles, aspect ratio, re-administration, mechanical property of polymer

## Abstract

The goal of this study is the preparation of safer coated microneedles so that tips remaining after the initial use are less likely to be reinserted on a second use. Twelve groups of uncoated microneedles (u-MNs) were prepared from the combination of three different aspect ratios (height to base width) and four kinds of polymer (polyethylene (PE), polypropylene (PP), nylon and polylactic acid (PLA)). After coating the u-MNs with polyvinyl alcohol formulation to make coated MNs (c-MNs), the force displacement of the u-MNs and the c-MNs was measured. The aspect ratio was reduced from 2.2, 2.5 and 3.0 with u-MNs to 1.3, 1.4 and 1.6 with c-MNs, respectively, after the coating formulation was applied to the MNs. All PLA MNs had a puncture performance of more than 95%. However, the puncture performance of u-MNs made of PE and of PP with a 3.0 aspect ratio was only 8% and 53%, respectively, whereas the rates of c-MNs made of PE and of PP were 82% and 95%, respectively. In animal experiments with PP MNs with a 3.0 aspect ratio, the 59% rate of puncture performance with u-MNs increased to above 96% with c-MNs and fell to 13% for r-MNs. Safe c-MNs can overcome the disadvantages of standard c-MNs by reducing the probable contamination of remaining tips after use. Safe c-MNs have advantages over standard c-MNs in terms of humidity resistance, reasonable cost, sterilization process and short processing time through the separate process of u-MN preparation and simple dip-coating.

## 1. Introduction

Microneedles (MNs) deliver active pharmaceutical ingredients (API) through the stratum corneum, the outermost layer of skin, regardless of molecular weight and API polarity [1,2,3,4]. There are four types of MNs: solid, coated, dissolving and swellable [5,6,7]. Coated MNs (c-MNs) are prepared by a simple process of coating the desired API formulation on the surface of polymer or metal uncoated microneedles (u-MNs) [8,9,10]. Water soluble MNs—made of water-soluble polymers have the advantage of no sharp biohazardous waste [11,12], but they have the limitation of rapid loss of mechanical strength at high relative humidity as well as biocompatibility issues related to the use of a water-soluble polymer [13,14,15,16]. c-MNs have excellent mechanical strength and are resistant to high humidity and moisture [6,17,18,19]. Thus, c-MNs can deliver drugs into the skin layer successfully regardless of environmental conditions [20,21,22,23]. However, the MNs remain after administration of c-MNs because only the coated formulation is dissolved in the skin and remained MNs can be reinserted [17,24,25]. The re-insertion of c-MNs is feasible, but cross-contamination from MNs has not been reported because the length of MNs is less than 1 mm and MNs have low-bioburden compared to the conventional syringe needle [26,27].

This study aims to develop safer c-MNs. Safe c-MNs, like standard c-MNs, will penetrate the skin only when the formulation is coated on the tips of u-MNs, and they will also feature the advantage of dissolving MNs, which cannot insert into skin after dissolution of tips. Skin puncture performance is controlled by the physical parameters of u-MNs, such as the mechanical properties of the polymer used for u-MNs and the aspect ratio of the tips [28,29,30]. When the drug formulation is coated on the tips of u-MNs, it forms a composite layer, which increases the mechanical strength of the c-MNs. However, the removal of composite layer added to u-MNs prevents the MNs from penetrating the skin after dissolution of the formulation in the skin, and thus the features of dissolving MNs are needed to provide safe c-MNs.

The puncture performance of MNs is controlled by the following four variables: (a) kind of polymer used—which determines the mechanical strength of the u-MNs; (b) aspect ratio of u-MNs tips; (c) addition of a coating layer; and d) dissolution of the coating layer of c-MNs. Puncture performance is measured according to these variables. In this study, in vivo skin puncture performance was conducted to compare with in vitro skin puncture performance.

## 2. Materials and Methods

### 2.1. Materials

Polylactic acid (PLA) was purchased from Green Chemical (Gyeonggi-do, South Korea). Polyethylene (PE) and polypropylene (PP) were purchased from Goodfellow (Huntingdon, UK). nylon and polyvinyl alcohol (PVA) were obtained from Sigma-Aldrich (St. Louis, MO, USA).

### 2.2. Fabrication of Microneedles

#### 2.2.1. Preparation of Uncoated Microneedles

Master structures of microneedles were fabricated by micro-milling of aluminum alloy with milling system (QuadMedicine, Inc., Seongnam, Korea). Polydimethylsiloxane (PDMS, Sylgard 184, Dow Corning, Midland, MI, USA) was poured into the microneedle master structures. The PDMS solution was cured for 3 h in an oven of 70 °C (BF-150C, Biofree, Seoul, Korea). The inner cavity of a polydimethylsiloxane (PDMS) mold was investigated using VHX-6000 digital microscope (KEYENCE, Osaka, Japan). Twelve kinds of u-MN arrays were manufactured using a micromolding process, with four groups of u-MNs made from four different polymers: polyethylene (PE), polypropylene (PP), nylon (N) or polylactic acid (PLA). Each group of u-MNs included three subgroups of different aspect ratios of height to width (HM/WM) (from the position 500 μm apart from tip base to tip apex): 2.2 (HM/WM: 300/135), 2.5 (300/118), and 3.0 (300/100) (see Table 1). Each MN array had 97 MNs arranged on a circular disk 1 cm in diameter. Pellets of PE, PP, nylon, and PLA were placed on a PDMS mold corresponding to each MN specification. The molding temperatures of the vacuum oven (VOS-301, EYELA, Tokyo) used to prepare the MNs were 170 °C, 190 °C, 180 °C and 195 °C for PE, PP, nylon and PLA, respectively, with the oven under 70 kPa vacuum. After being prepared, all samples were treated using a UV-ozone treatment machine to promote wetting with a hydrophilic coating solution.

#### 2.2.2. Dip-Coating of Microneedles

Twelve kinds of c-MN arrays were manufactured using a dip-coating process. The coating solution had 20% (w/w) of polyvinyl alcohol (PVA) in distilled water. The coating solution was loaded into the coating well to a depth of 600 μm. The ozone-treated uncoated microneedles were dipped into the reservoir at a rate of 20 mm/s in the coating reservoir. Coated samples were dried at room temperature. Samples were coated with platinum and then the geometries of the samples were observed using scanning electron microscopy (SEM). Thirty-six MNs (12 u-MNs, 12 c-MNs, and 12 r-MNs) are shown in Table 1. For c-MNs, the ratio of HM/WM was defined as “distance from convex to tip/diameter in the convex portion of the solidified coating formulation.” r-MNs were microneedles with coated formulation removed by dissolution in skin after insertion.

### 2.3. Mechanical Properties of Polymer

In order to measure the mechanical strength of PE, PP, nylon and PLA used in this study, specimens of each polymer were prepared in accordance with ASTM D638 Type 1 (for tensile strength) and ASTM D790 (for flexural strength). The fabricated specimens were measured for flexural strength, tensile strength and tensile modulus using the universal testing machine (Zwick / Roell Z020, Ulm, Germany).

### 2.4. In Vitro Penetration Test

Evaluation of in vitro puncture performance was carried out for each group of MNs described in Table 1 using porcine skin. For u-MNs, MNs were applied to the full thickness of porcine skin (CRONEX, Seoul, South Korea) for 10 s with 30 N of force and 20 μL of trypan blue solution was applied on the treated skin for 10 min. The number of resulting blue dots after removal of the excess dye solution were counted using an optical microscope (sv-35, Sometech, South Korea). The puncture performance was defined by comparing the number of blue dots to the total number of MNs. For the group of c-MNs with the coating formulation on u-MNs, c-MNs were fixed with clamp of 3–4 kg/cm^2^ for 30 min after pressing them with the fingers for 10 s, and then trypan blue solution was applied on the treated area in the same way to check the puncture performance. After insertion and removal of the c-MNs, r-MNs were re-administered into the porcine skin. The dyeing process and the calculation process were repeated for the reinserted r-MNs. The means and standard deviations were calculated for three samples in each group.

### 2.5. Force–Displacement Test of Microneedles

The force displacement of the MNs was observed using a uniaxial force analyzer (500-N Zwicki, Zwick GmbH & Co. KG, Ulm, Germany). Force–displacement curves were obtained by applying force to four MNs on an array for each sample until the force of 2 N was reached.

### 2.6. In Vivo Puncture Performance Test of PP Microneedles

Evaluation of in vivo puncture performance with u-H-PP MNs, c-H-PP MNs and r-H-PP MNs was conducted in the same way as in vitro puncture performance, as explained in Section 2.4. Six to seven-week-old BALB/c mice (Orient Bio, Seoul, South Korea) were housed in a specific pathogen-free facility. The mice were provided ad libitum access to sterilized food and water. All animal protocols were approved by the Institutional Animal Care and Use Committee of the Yonsei College of Medicine (IACUC #2019–0175, Seoul, South Korea). All experiments were performed under anesthesia via injection of a mixture of Zoletil 50 mix (0.015 mL/20 g; Virbac, Carros, France) and Rompun (0.5 mg/20 g; Bayer Korea, Seoul, South Korea). The u-H-PP MNs were pressed on the back of the mice with fingers for 10 s after shaving. The c-H-PP MNs were pressed with the thumb and then the MNs were attached using an adhesive plaster (Dermaplast, IVF Hartmann, Neuhausen, Switzerland) and clamped with 3–4 kg/cm^2^ for 30 min. Trypan blue solution was applied on the treated skin for 10 min to stain the holes generated by successful puncture. Puncture performance was carried out in the same manner for the r-H-PP MNs. Images of stained holes in the skin were taken using a camera (Digibird, Seoul, South Korea) and puncture performance was calculated for samples.

## 3. Results and Discussion

### 3.1. Geometries of Three Subgroups of Microneedles

The u-MNs were prepared by molding polymer; four kinds of polymer can be replicated from a mold after melting. As a medical device of u-MNs, polymer MNs can be mass produced, easily sterilized and made ready-to-use [31,32]. Thus, u-MNs have the advantages of the economical price of polymer, manufacture with various polymers, simplicity of the molding process, ease of sterilization and coatings of various API.

One of the critical concerns regarding polymer MNs is their mechanical stability. The mechanical failure of the MNs was determined by the aspect ratio of height to base width, Young’s modulus and the tensile strength of the polymer [4,28,33]. The deformation pattern of the MNs was also determined by the above variables. Therefore, the puncture performance of MNs was able to be designed by manipulating these variables.

Twelve kinds of u-MNs with three kinds of aspect ratio were prepared from four kinds of polymer (PE, PP, nylon and PLA). All MNs have the same height (800 μm). The u-MNs had different widths (135 μm, 118 μm and 100 μm) at 300 μm from the top and different aspect ratios (2.2, 2.5 and 3.0, respectively), as shown in illustrations 1–3 of Table 2. The change in each group of u-MNs after the coating process is shown in Table 2. The coating amount was the same for all samples (200–300 μg per MN). When coating a structure having a 2.5 aspect ratio, the coating thickness is about 35 μm, regardless of the kind of polymer used, because of the surface treatment with ozone plasma.

The maximum depth of the coating solution could be 600 μm, but the actual coating depth did not exceed 500 μm, due to the surface property of the polymer and the high viscosity of the polyvinyl alcohol solution. In the case of u-M-MNs and u-H-MNs, which was step pyramid, it was difficult for the coating solution to pass the step due to the surface tension generated at the edge of the step. Therefore, for all u-MNs, the solution was coated up to a distance of 500 μm from the tip end of the microneedles. Inkjet printing technology was utilized to coat metal microneedles with the drug formulation, and the formulation was distributed across the surface of fully coated needles [34]. However, only a formulation with low viscosity can be coated, and the coating status is affected by the distance from the nozzle or the spray angle of the nozzle. The dip-coating method can be used for coating a solution that has a wide range of viscosity. However, the actual coating depth can differ from the depth of the coating solution due to the interfacial tension between the coating solution and the microneedle material and viscoelastic property of coating solution.

**Figure 1:**
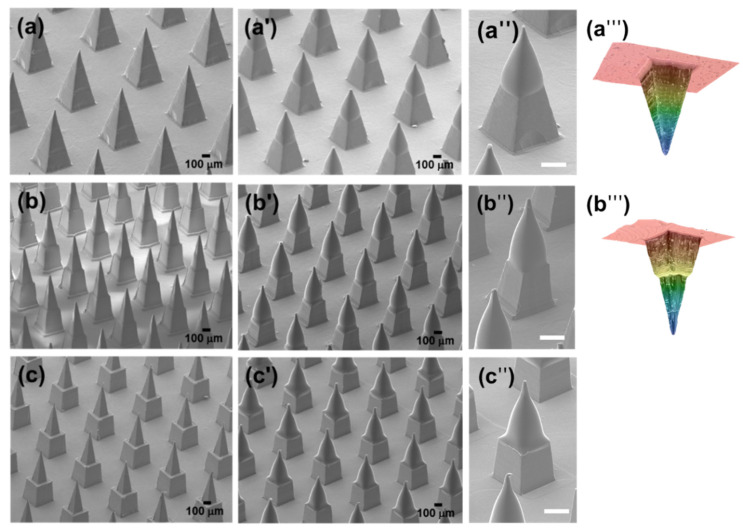
SEM image of u-MNs with (**a**) aspect ratio 2.2; (**b**) aspect ratio 2.5; (**c**) aspect ratio 3.0; c-MNs with (**a’**) aspect ratio 1.3; (**b’**) c-MNs aspect ratio 1.4; (**c’**) c-MNs aspect ratio 1.6; Magnified image of c-MNs with (**a’’**) aspect ratio 1.3; (**b’’**) aspect ratio 1.4; (**c’’**) aspect ratio 1.6. White scale bar means 200 μm. Laser microscopic image of microneedle cavity of mold of (**a’’’**) u-L-MNs (aspect ratio 2.2) and (**b’’’**) u-M-MNs (aspect ratio 2.5).

The information on the geometries of the samples is summarized in Table 2. As shown in Figure 1, the aspect ratios of u-MNs changed from 2.2, 2.5 and 3.0 before coating to 1.3, 1.4 and 1.6 after coating. When the coating material had dissolved after the c-MNs were inserted into the skin, the c-MNs returned to their initial aspect ratio.

### 3.2. Mechanical Properties of Material

Tensile modulus and flexural strength of PE, PP, nylon, and PLA polymers used as MN material in this study were measured. As shown in Table 3, tensile modulus was 1.1, 1.0, 1.2 and 2.1 GPa for PE, PP, nylon and PLA, respectively. As shown in Table 3, flexural strength was about 22, 31, 45 and 85 MPa for PE, PP, nylon and PLA, respectively. Tensile modulus means the rigidity of the polymer [35]. PE, PP and nylon were similar in terms of rigidity, but nylon was higher than PE and PP in mechanical strength when also considering flexural strength. PLA had the highest value for both rigidity and strength.

The mechanical properties of each group of MNs were evaluated by force–displacement pattern analysis. The slope of the force displacement increased significantly after coating in all kinds of MNs, as shown in Table 4. After coating, the aspect ratio was reduced and the coating formulation formed a composite layer to have the improved mechanical strength and brittleness suitable for skin penetration. After coating the formulation on the u-MNs, the mechanical strength was increased and at the same time the hardness of the MN tip was increased. The increased mechanical strength of the MNs that was attained by adding the coating abated after the coating formulation was dissolved in the skin, lowering the puncture performance of MNs.

### 3.3. In Vitro Puncture Performance

**Figure 2:**
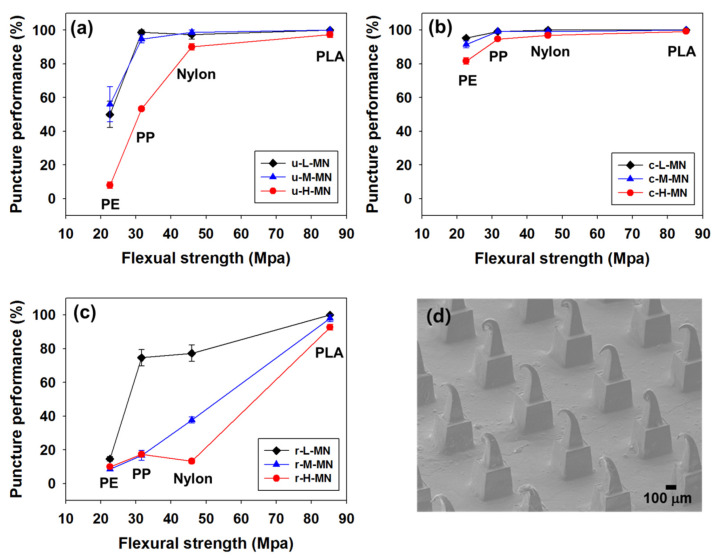
Puncture performance of (**a**) u-MNs, (**b**) c-MNs, (**c**) r-MNs MNs according to the mechanical strength of the polymer (polyethylene (PE), polypropylene (PP), nylon and polylactic acid (PLA)) and the aspect ratio of the MNs, (**d**) scanning electron microscope image of r-H-N MNs with bent tips. Flexural strengths of PE, PP, nylon and PLA are 22, 31, 45 and 85 MPa, respectively.

As shown in Figure 2 and Table 5, puncture performance was evaluated in porcine skin in vitro for u-MNs and c-MNs according to the mechanical strength of the polymer and the aspect ratio of the MNs. The x-axis in Figure 2 represents flexural strength and the flexural strengths of PE, PP, nylon and PLA are 22, 31, 45 and 85 MPa, respectively. The polymers of PE, PP, nylon and PLA are shown on the x-axis according to their flexural strength. As shown in Figure 2a, the uncoated PLA MNs showed over 95% puncture performance for all aspect ratios. However, as shown in Figure 2a, for aspect ratio 3.0, puncture performance of u-MNs decreased as the mechanical strength of the polymer decreased (in order: PLA, nylon, PP and PE). The puncture performance of PE with the 3.0 aspect ratio was less than 10%, and that of PP with the 3.0 aspect ratio was only 50%. The puncture performance of the MNs can be controlled by the selection of the kind of polymer and the aspect ratio of u-MNs. A comparison of Figure 2a with Figure 2b shows that overall puncture performance increased after the coating layer was added. When c-MNs was formed on the u-MNs by adding PVA coated layer, the aspect ratio was reduced and the u-MNs became mechanically more stable, resulting in greater penetration into the skin (over 95% puncture performance).

The c-MNs were applied to the porcine skin in vitro at a pressure of 3–4 kg/cm^2^ for 30 min and then removed. A range of force from 1 kg/cm^2^ to 4 kg/cm^2^ was used to conduct the clinical study of MNs; this range can be generated by thumb pressure [38,39]. In another study measuring the insertion depth of the MNs using a pressure-indicating film sensor, 2–5 kg of force was applied to the MNs, and manual thumb pressure was characterized by a force of about 3–4 kg [40,41]. When pressing the MNs with manual finger pressure, it is important to apply a force above the determined force and feedback on the applied force [38,39]. The force applied to the MNs in this study was in the range of 3–4 kg. The low puncture performance after use was determined by the increase of the aspect ratio and removal of the coated layer by the dissolution of the coated formulation after the c-MNs were inserted into the skin. When c-MNs return to being u-MNs after the coating formulation is dissolved (r-MNs), the skin puncture performance of r-MNs should be similar to that of the u-MNs. However, as shown in Figure 2a,c, the skin puncture performance of r-MNs was lower than that of u-MNs. After the c-H-MNs were applied to the skin for 30 min, the MN tips were bent and deformed by the force applied to the tips during insertion, as shown in Figure 2d. When the MN tips were pressed on the hard surface beneath the porcine skin after insertion, the tips were bent, resulting in failure of re-insertion into the skin. It was therefore recommended to apply a force of 3–4 kg/cm^2^ on the skin close to bone to induce deformation of tips in order to prevent re-administration. In this study, MNs were pressed onto the full thickness of porcine skin placed on a hard surface with a force in the range of 3–4 kg/cm^2^. In a clinical study, MNs were pressed against the wrist, whose skin was close to bone [42]. If MNs encountered a hard surface after administration, the tips were deformed, which made it difficult to penetrate the skin after use. Therefore, all r-MNs with a 3.0 aspect ratio (r-H MNs) except r-H-PLA MNs showed significantly lower (below 20%) puncture performance.

**Figure 3:**
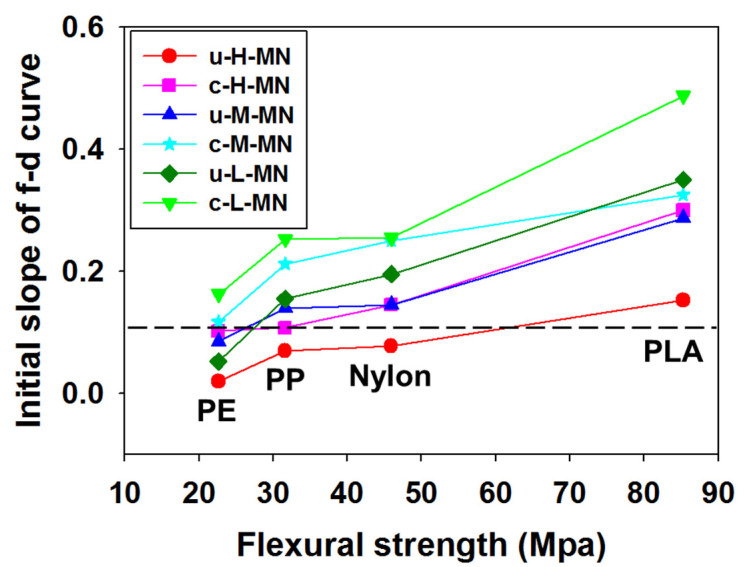
Relation of penetration performance to (1) initial slope of force and displacement, (2) kind of polymer (polyethylene (PE), polypropylene (PP), nylon and polylactic acid (PLA)) and (3) aspect ratio of 3.0, 2.5 and 2.2 for u-MNs and 1.6, 1.4 and 1.3 for c-MNs. Dashed line indicates the 95% puncture performance. Flexural strengths of PE, PP, nylon and PLA are 22, 31, 45 and 85 MPa, respectively.

For each group of MNs, the relationship between the initial force–displacement slope and puncture performance was shown according to the polymer type in Figure 3. In the case of the microneedles used in this study, the slope of the initial force–displacement is significant because the value of slope determines the strength of the tip. The comparison of the mechanical strength between materials and the aspect ratio of the microneedle structure can be obtained through the initial slope value. Puncture performance of 95% is shown by the dotted line. Above the dotted line, more than 95% of MNs penetrated. Puncture performance can be anticipated by the mechanical properties of polymer, aspect ratio of u-MNs and the existence of coating formulation. The initial force–displacement slope of MNs is a characteristic representing the initial deformation of the tip of MNs and it can be used as criteria for predicting the puncture performance of the MNs. In Figure 3, an initial slope of 0.1 corresponded to a puncture performance of 95%. The initial slope indicates the elastic deformation of the MN tips, and the hardness of the tips played an important role in puncture performance. The puncture performance of PP was sensitive to the variables and the 95% puncture performance of polymer depended on coating and aspect ratio. For example, the puncture performance of u-H-PP was low (53% ± 1%), but the puncture of c-H-PP was more than 95% after coating. Figure 3 shows that the mechanical strength of the macroscopic properties of polymer and force-displacement pattern analysis of the MNs can be used to control puncture performance according to the aspect ratio of u-MNs. By reducing puncture performance as described above, it was possible to minimize the problem of contamination caused by the re-administration of r-MNs.

### 3.4. In Vivo Puncture Performance

**Figure 4:**
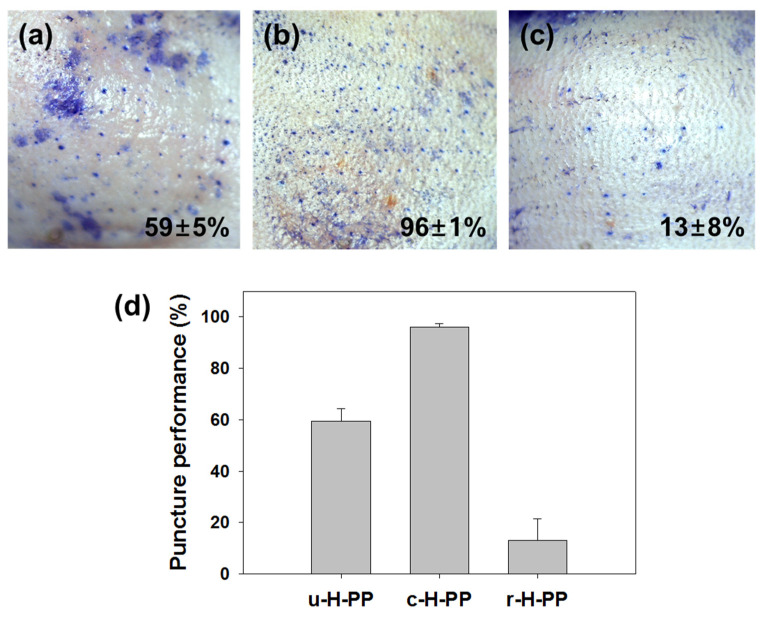
Image of in vivo mouse skin stained with trypan blue after insertion and removal of (**a**) u-H-PP-MNs (uncoated microneedles with high aspect ratio), (**b**) c-H-PP-MNs (coated microneedles with high aspect ratio) and (**c**) r-H-PP-MNs (microneedles with coated formulation removed with high aspect ratio); (**d**) in vivo puncture performance comparison of u-H-PP, c-H-PP and r-H-PP.

Based on the in vitro experiments, skin puncture was performed on mice in vivo to confirm whether the results obtained from the in vitro study were applicable to the in vivo experiment. Among the various polymers, PP was selected because puncture performance was greatly affected by the aspect ratio change occurring before and after coating and after dissolution of the coating formulation. The MNs used in the in vivo experiments were u-H-PP, c-H-PP and r-H-PP. As shown in Figure 4, in vivo results showed skin puncture performance of 59% ± 5%, 96% ± 1% and 13% ± 8% for u-H-PP MNs, c-H-PP MNs, and r-H-PP MNs, respectively. This result confirmed that the in vitro results were used to predict in vivo results. The u-H-PP MNs showed a skin puncture rate of about 59% due to their low mechanical stability, but c-H-PP MNs after coating of u-H-PP MNs showed a skin puncture rate of 96%, meaning that the coated formulation was successfully delivered into the skin layer. The application time of the c-H-PP MNs was 30 min and was continuously applied with approximately 3–4 kg/cm^2^ of force. When the MNs were removed after 30 min of administration and then re-administered, the skin puncture performance dropped to 13%, thereby minimizing the chance of contamination. The 13% rate of r-H-PP MNs was lower than that of u-H-PP MNs because deformation occurred at the tips of the MNs during insertion. Therefore, in general, when a hard tissue such as a wrist bone was encountered after MNs were pushed into wrist skin (a procedure used in vaccine MN administration in the clinical study), the MN tips were deformed and thus the puncture performance was drastically reduced.

## 4. Conclusions

The aim of this study was to develop safer coated MNs that reduce the occurrence of punctures in the skin after the MNs are used. Before a solid formulation was coated to u-MNs, physical parameters were found that were not sufficient for the MNs to penetrate the skin. However, adding the coating formulation increased the mechanical strength of the MNs, thereby improving the puncture performance and achieving the desired drug delivery. Then, the dissolution of the coating formulation in the skin after insertion lessened the mechanical stability of the MNs and the rate of skin puncture was lowered as a result.

The newly introduced safe c-MNs have the advantages of both c-MNs and dissolving MNs. Safe c-MNs are not affected by moisture, and they can deliver a variety of API regardless of environmental condition. Safe c-MNs leave no biohazardous residue like dissolving microneedles, and the low-temperature process can be used to load thermally sensitive API on the c-MNs. Safe c-MNs are a microneedle system that can be safely and effectively used for the delivery of various vaccines and drugs.

## Figures and Tables

**Table 1:** Definition of samples based on the coating status, aspect ratio and kind of polymer used. Coating status (u—uncoated; c—coated; r—coated formulation removed); Aspect ratio of u-MNs and c-MNS (L—low 2.2/1.3 (u/c); M—medium 2.5/1.4 (u/c); H—high 3.0/1.6 (u/c)), Polymer (PE—polyethylene; PP—polypropylene; N—nylon; PLA—polylactic acid).

Aspect Ratio	PE	PP	nylon	PLA
L (2.2/1.3:(u-coated/coated))	u-L-PE	u-L-PP	u-L-N	u-L-PLA
	c-L-PE	c-L-PP	c-L-N	c-L-PLA
	r-L-PE	r-L-PP	r-L-N	r-L-PLA
M (2.5/1.4:(u/c))	u-M-PE	u-M-PP	u-M-N	u-M-PLA
	c-M-PE	c-M-PP	c-M-N	c-M-PLA
	r-M-PE	r-M-PP	r-M-N	r-M-PLA
H (3.0/1.6:(u/c))	u-H-PE	u-H-PP	u-H–N	u-H-PLA
	c-H-PE	c-H-PP	c-H–N	c-H-PLA
	r-H-PE	r-H-PP	r-H–N	r-H-PLA

**Table 2:** Information on the geometries of three aspect ratio of microneedles (MNs) before and after coating. HM—height; WM—width.

MNs	WM (μm)	HM (μm)	Aspect Ratio (HM/WM)	Angle θ (°)	Figure 1
(1) u-L (uncoated-low aspect ratio)	135	300	2.2	77	(a)
(2) u-M (uncoated-medium aspect ratio)	118	300	2.5	79	(b)
(3) u-H (uncoated-low aspect ratio)	100	300	3.0	81	(c)
(4) c-L (coated u-L)	225	300	1.3	69	(a’)
(5) c-M (coated u-M)	214	300	1.4	70	(b’)
(6) c-H (coated u-H)	188	300	1.6	73	(c’)
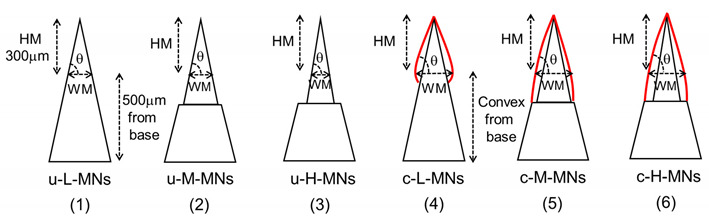					

**Table 3:** Mechanical properties of four polymers used in the study: polyethylene (PE), polypropylene (PP), nylon 12 (nylon), polylactic acid (PLA) (unit: MPa).

Mechanical Property	PE	PP	Nylon	PLA
Tensile modulus	1100	1000	1200	2100
Flexural strength	22	31	45	85
Elongation ratio (%)	500 [35]	100 [36]	90 [36]	4.3 [37]

**Table 4:** Initial slope of force displacement of MNs before and after coating formulation with respect to kind of polymer (PE, PP, nylon and PLA) and aspect ratio.

MNs	Aspect Ratio	PE	PP	N	PLA
u-MN	u-L (2.2)	0.0525	0.155	0.195	0.35
	u-M (2.5)	0.085	0.14	0.145	0.2875
	u-H (3.0)	0.02	0.07	0.0775	0.1525
c-MN	c-L (1.3)	0.1625	0.2525	0.255	0.4875
	c-M (1.4)	0.1175	0.2125	0.25	0.325
	c-H (1.6)	0.1025	0.1075	0.145	0.3

u—uncoated, c—coated, L—low aspect ratio, M—medium aspect ratio, H—high aspect ratio, PE polyethylene, PP—polypropylene, N—nylon; PLA—polylactic acid.

**Table 5:** Puncture performance of MNs according to the mechanical strength of the polymer (polyethylene (PE), polypropylene (PP), nylon (N), polylactic acid (PLA)) and the aspect ratio.

MNs	Aspect Ratio	PE	PP	N	PLA
u-MN	2.2	50 ± 13	99 ± 2	97 ± 5	100
	2.5	56 ± 18	95 ± 4	99 ± 2	100
	3.0	8 ± 3	53 ± 1	90 ± 3	97 ± 3
c-MN	1.3	95 ± 2	99 ± 2	100	100
	1.4	91 ± 5	99 ± 1	99 ±2	100
	1.6	82 ± 4	95 ± 1	97 ± 2	99 ± 1
r-MN	2.2	15 ± 2	75 ± 8	77 ± 8	100
	2.5	9 ± 1	17 ± 5	38 ± 3	98 ± 3
	3.0	6 ± 5	17 ± 2	13 ± 2	93 ± 3

## Abbreviations

MNs	microneedles
API	active pharmaceutical ingredients
u-MNs	uncoated microneedles
c-MNs	coated microneedles
r-MNs	microneedles with coated formulation removed
u-L-MNs	uncoated microneedles with low aspect ratio
u-M-MNs	uncoated microneedles with medium aspect ratio
u-H-MNs	uncoated microneedles with high aspect ratio
c-L-MNs	coated microneedles with low aspect ratio
c-M-MNs	coated microneedles with medium aspect ratio
c-H-MNs	coated microneedles with high aspect ratio
r-L-MNs	microneedles with coated formulation removed with low aspect ratio
r-M-MNs	microneedles with coated formulation removed with medium aspect ratio
r-H-MNs	microneedles with coated formulation removed with high aspect ratio
